# Risk factors for hip fracture in New Zealand older adults seeking home care services: a national population cross-sectional study

**DOI:** 10.1186/s12877-019-1107-1

**Published:** 2019-03-25

**Authors:** Rebecca Abey-Nesbit, Philip J. Schluter, Tim Wilkinson, John Hugh Thwaites, Sarah D. Berry, Hamish A. Jamieson

**Affiliations:** 1 Christchurch School of Medicine and Health Sciences, University of Otago, Christchurch, New Zealand; 2 School of Health Sciences, University of Canterbury–Te Whare Wānanga o Waitaha, Christchurch, New Zealand; 3 School of Clinical Medicine – Primary Care Clinical Unit, The University of Queensland, Brisbane, Australia; 4 Canterbury District Health Board, Christchurch, New Zealand; 5 Harvard Medical School, Boston, MA USA; 6 Division of Geriatric Medicine, Department of Medicine, Beth Israel Deaconess Medical Center, Boston, MA USA; 7 Hebrew Senior Life, Boston, MA USA

**Keywords:** Hip fracture, New Zealand, interRAI, Older people, Fracture risk

## Abstract

**Background:**

Hip fractures are a common injury in older people. Many studies worldwide have identified various risk factors for hip fracture. However, risk factors for hip fracture have not been studied extensively in New Zealand. The interRAI home care assessment consists of 236 health questions and some of these may be related to hip fracture risk.

**Methods:**

The cohort consisted of 45,046 home care clients aged 65 years and older, in New Zealand. Assessments ranged from September 2012 to October 2015. Hip fracture diagnosis was identified by linking ICD (International Classification of Diseases) codes from hospital admissions data (September 2012 to December 2015) to the interRAI home care data. Unadjusted and adjusted competing risk regressions, using the Fine and Gray method were used to identify risk factors for hip fracture. Mortality was the competing event.

**Results:**

The cohort consisted of 61% female with a mean age of 82.7 years. A total of 3010 (6.7%) of the cohort sustained a hip fracture after assessment. After adjusting for sociodemographic and potentially confounding variables falls (SHR (Subhazard Ratio) = 1.17, 95% CI (Confidence interval): 1.05–1.31), previous hip fracture (SHR = 4.16, 95% CI: 2.93–5.89), female gender (SHR = 1.38, 95% CI: 1.22–1.55), underweight (SHR = 1.67, 95% CI = 1.39–2.02), tobacco use (SHR = 1.56, 95% CI = 1.25–1.96), Parkinson’s disease (SHR = 1.45, 95% CI: 1.14–1.84), and Wandering (SHR = 1.36, 95% CI: 1.07–1.72) were identified as risk factors for hip fracture. Shortness of breath (SHR = 0.80, 95% CI: 0.71–0.90), was identified as being protective against hip fracture risk. Males and females had different significant risk factors.

**Conclusions:**

Risk factors for hip fracture similar to international work on risk factors for hip fracture, can be identified using the New Zealand version of the interRAI home care assessment.

**Electronic supplementary material:**

The online version of this article (10.1186/s12877-019-1107-1) contains supplementary material, which is available to authorized users.

## Background

Hip fractures cause significant disability for many older adults, with some of the worst outcomes for those aged 65 years or older [1]. Having a hip fracture in later life can lead to long recovery periods, a decreased quality of life, higher mortality rates and increased likelihood of entry into aged residential care (ARC) compared to those without a hip fracture [1–3].

Improving health outcomes using health data is a priority for the New Zealand government [4]. Since 2012, all community dwelling older New Zealanders requiring home health care services undergo a standardised assessment using the interRAI home care (interRAI-HC) assessment. At that time, New Zealand became one of the first countries in the world to mandate a standardised comprehensive medical and functional assessment for all older people who are seeking home care services. The interRAI-HC assessment is a comprehensive clinical assessment with questions on 20 health and social domains including disease diagnoses, cognitive function and social relationships. The interRAI-HC assessment is used to assess the health needs of frail older people and people with complex needs, living within the community. Typical home health care services being sought include visiting nurses, access to falls prevention programmes, and meals. In 2013 there were approximately 607,032 adults aged 65+ years in New Zealand [5]. A large number of these people have undergone a home care assessment, and as the ageing population increases many more people will also require health services. Using health data to identify individuals at greatest risk for hip fracture may facilitate prevention and delay adverse outcomes.

There have been many studies conducted identifying hip fracture risk in older adults. These include a variety of different cohorts including those recently admitted to hospital, those living in ARC facilities, and those living within the community. Each cohort has different risk profiles as they are subject to different environments and have differing health needs [6–9]. Stolee et al. (2009) identified risk factors for hip fracture in community dwelling older people requiring home care [7]. Their work used the Minimum Dataset (MDS), a precursor to the interRAI homecare assessment, and was based on 40,279 Canadian participants. Risk factors identified by Stolee et al. for a home care population were older age, females, osteoporosis, falls, unsteady gait, tobacco use, malnutrition and cognitive impairment [7]. Other studies identifying hip fracture risk for older people in older cohorts found factors such as differing ethnic backgrounds, bone mineral density (BMD), previous hip fractures, reduced physical activity, body mass index (BMI), chronic health conditions, and medications to be significant [2, 8, 10–16].

Hip fractures are a worldwide issue and have been studied extensively, yet there has been very little research in New Zealand on hip fracture risk. There is one known paper addressing hip fracture incidence rates in Māori (New Zealand’s indigenous peoples) and non-Māori people [17]; and the study found that non-Māori were at a higher risk than Māori – but that the incidence rates were increasing over time for both ethnic groups. However, this paper was published in 1995, there has been significant population change, and changes in prevention and health service delivery since that time. It is likely the risk profile and possible risk factors have also changed. Another study explored whether urinary incontinence was an independent risk factor for falls and hip fractures in community dwelling older men and women with complex needs. After controlling for confounders, results showed that urinary incontinence was not an independent risk factor for hip fracture [18]. Two further studies, identified specific medications were associated with an increased risk of fracture among older New Zealanders [14, 19]. No recent studies have specifically sought to identify the suite of risk factors associated with hip fractures amongst older adults in New Zealand as a group, and for the important ethnic groups.

There are methodological limitations with many previous studies of hip fracture risk. Studies have used an array of different statistical techniques, predominantly regression models such as Poisson, multivariate logistic regression, or Cox proportional hazards models [7, 20, 21]. However, due to the non-negligible likelihood of death amongst older adults with complex needs, more recent studies have employed competing risk regression models as they provide a less biased estimate of risk factors. [22, 23]. For example, Berry et al. (2017) recently determined risk factors for hip fracture and created a hip fracture risk score based on nursing home residents derived from a competing risk regression analysis [10].

The primary objective of this study was to determine risk factors for hip fracture in older people living in the community in New Zealand and receive home care. A secondary objective was to identify whether there were differences in risk factors for hip fracture between males and females.

## Methods

### Study design

This study used a time-to-event analysis from a national cohort.

### Participants

People aged 65+ years living in the community with an interRAI-HC assessment undertaken between 1 September 2012 and 31 October 2015, who consented to their data being used for planning and research purposes. Only the first assessment for all participants were used, any subsequent assessments were removed from analysis. Individuals with end-stage disease, six or fewer months to live, as noted in the interRAI-HC assessment were excluded. Within the interRAI-HC assessment there is a question on living arrangements at time of assessment and a small number of participants had indicated living in a long-term care facility at time of assessment. These people were excluded from analysis along with anyone admitted to an ARC facility within 30 days of their assessment as this study was looking only at those living in the community in non-residential care. Similar to the work carried out by Berry et al. [10], 22,291 participants were randomly omitted from this study, as forthcoming work will build a prediction model based on these results and validate it against those randomly excluded.

### Instruments/variables

The interRAI-HC version 9.1 (© interRAI corporation, Washington, D. C., 1994–2009) assessment tool is a comprehensive geriatric assessment record consisting of 236 questions across 20 domains such as cognitive function, nutrition, disease diagnoses and psychosocial well-being [24, 25]. The home care assessment is used for all older people requiring publically funded long-term home care services or aged residential care admission. Patients are referred by health practitioners to have their needs assessed by a trained interRAI assessor. Assessors visit the person in their own home and use a variety of sources to complete each assessment, including observations, interviews with the individual and their family members, and medical records. Assessors undergo rigorous training and are reassessed annually to ensure each assessor meets interRAI standards. All data are entered into an electronic database, which are collected and maintained by New Zealand’s Technical Advisory Services (TAS). Participant consented data (approximately 93% of all assessments undertaken) are released by TAS with the approval of the Ministry of Health. The assessment is also used to aid in the planning of home support and health care [24]. All questions are recorded electronically and assessments are linked with the National Health Index (NHI) number. The NHI is a unique identifier given to anyone in New Zealand receiving health services. The NHI can be encrypted and linked with other health datasets such as mortality information and hospital admissions within New Zealand. Variables of interest were identified from the literature and recoded within the interRAI-HC data. Variables were obtained from several domains within the interRAI dataset such as: Demographics, Cognitive/Functional, Falls and Fractures, Neuropsychiatric, Pain, Nutrition, Co-morbidities. A full list of the variables of interest, used for analysis can be found in Additional file 1: Table S1. Previous studies have highlighted age, sex and ethnicity as known demographics relating to hip fracture risk. Ethnicity was classified as Māori, Pasifika, Asian, European and Other. Participants were given the option to choose up to three ethnic identities and priority coding was used during the data cleaning process to reduce this to a single ethnicity. Where participants indicated more than one ethnicity priority was given to Māori, then Pasifika, and then Asian ethnicities. Age, sex, and ethnicity were employed as potential confounders when creating the adjusted model.

Hip fracture data were obtained from the National Minimum Dataset (NMDS) [26], released from the Ministry of Health with encrypted NHI numbers for all interRAI-HC participants who consented. Hip fractures were identified using ICD-10-AM (International Statistical Classification of Diseases and Related Health Problems, Tenth Revision, Australian Modification) diagnostic codes S720, S721, S722, S723, S724, S728, and S729. The first instance of hip fracture after an individual’s assessment was used. All hip fractures occurred before or on the 31 October 2015.

Mortality data were obtained from the Mortality Collection (MORT) [27], and were released from the Ministry of Health with encrypted NHI numbers for all interRAI-HC participants who consented. All deaths occurred before or on the 31 October 2015.

### Statistical analysis

Reporting of analyses conformed to STROBE STrengthening the Reporting of OBservational studies in Epidemiology and RECORD (REporting of Studies Conducted using Observational Routinely collected Data) guidelines [28] to ensure this study reports results accurately and clearly. Basic frequency distributions of each variable of interest were examined as a total of the population. Competing risk regression models, using the Fine and Grey method [29], were utilised where hip fracture was the failure event and death was the competing event. Crude models were individually conducted for variable of interest and then a fully adjusted model which included age, sex and ethnicity was undertaken. All variables in the unadjusted models were included in the adjusted model. Subhazard ratios (SHRs) and 95% confidence intervals (CIs) were reported for each variable of interest. The data was stratified by sex to assess risk profiles for both males and females and adjusted competing risk models were undertaken. IBM SPSS version 23 [30] was used for general analyses and data cleaning, and Stata SE version 14.1 [31] was used to run competing risk regressions, and α = 0.05 defined statistical significance.

### Ethics

Permission for this study was approved by the Ministry of Health’s Health and Disability Ethics Committees (14/STH/140) and only includes anonymised data provided by individuals who consented to their information being used for planning and research purposes.

## Results

### Participants

**Fig. 1:**
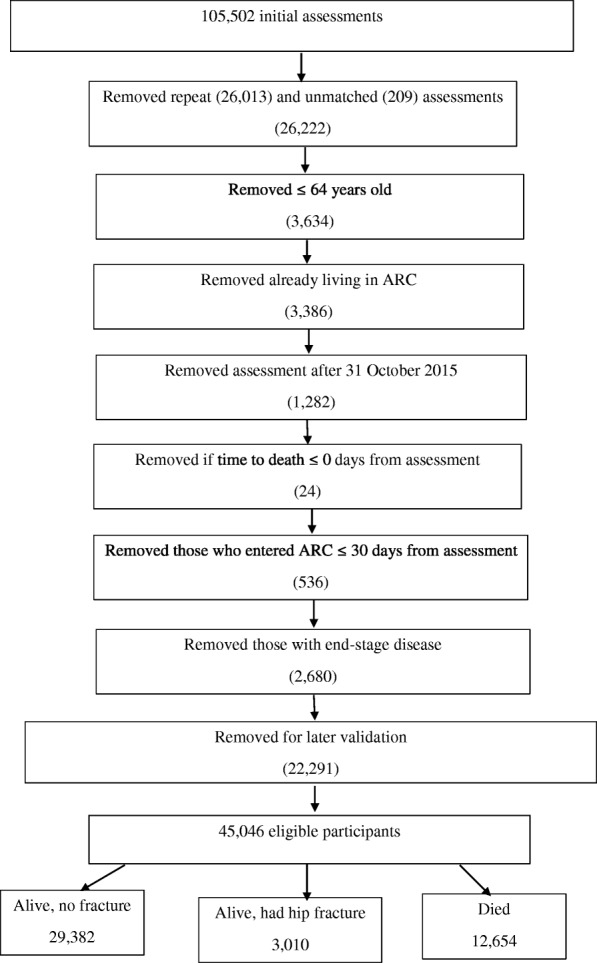
Exclusion criteria for interRAI HC assessments

After applying the exclusion criteria, the sample consisted of 45,046 participants. Figure 1 below, details the exclusion criteria. Note, 22,291 were randomly omitted for this study, as forthcoming work will build a prediction model based on these results and validate it against those randomly excluded.

### Demographics

The mean age at assessment was 82.7 years (range: 65 to 106 years). Overall, there were 27,705 (61.5%) females and 17,339 (38.5%) males. Comparatively, according to the New Zealand 2013 census of those aged 65+ years in New Zealand there were 54.1% female and 45.9% males [5]. Within the cohort 88.2% of people identified as European these are similar to 2013 census information. However, Pasifika make up 3.2% (1430) and Asians make up 2.3% (1037) of the cohort compared to 2.3 and 4.7%, respectively, of New Zealanders aged 65+ years [5]. Approximately, 2% (918) of individuals had no medications prescribed to them, compared to 98% (44,125) individuals who stated they had some medications prescribed by a physician. Adherence to medications varied with 83.7% of people stating they always adhered to the medications prescribed, 11.5% were adherent 80% of the time, and 2.7% were adherent less than 80% of the time, including failing to purchase prescribed medications. 47.5% (21,739) of individuals had a cognitive performance scale (CPS) of 2 or higher indicating they had some degree of cognitive impairment. A CPS from 2 to 6 indicates mild to very severe cognitive impairment [32].

### Fractures and deaths

Of the test cohort 3010 (6.7%) sustained a hip fracture, 12,654 (28.1%) had died and 29,382 (65.2%) had not encountered either event by the end of the study period. Median follow-up time after the interRAI-HC assessment was 13.9 months (25th percentile 5.3 months, 75th percentile 23.8 months) from assessment, with a total person-time of 55,444 years.

### Unadjusted and adjusted analyses

The SHR for females, in the adjusted model is 1.38 suggesting that females have a higher risk of hip fracture than males. The 85–94 year-old age group had the highest percentage of hip fractures (50.1% of all hip fractures) and there was a slight increase in hip fracture incidence rates with age. The SHR for each age group shows that as age increases the risk of sustaining a hip fracture increases (as shown in Table 1). The majority of the cohort are European ethnicity (88.2%) and most hip fractures were sustained by those of European ethnicity (94.1%). Table 1 below, outlines basic descriptive information for each of the demographic variables and shows the crude and adjusted SHRs.

**Table 1:** Demographic details

		First Event				
Variable names	Total *n* (%)	Alive, no fracture	Fracture	Death	Unadjusted	Adjusted
Sex^a^						
Male	17,339 (38.5)	10,449 (35.6)	907 (30.1)	5983 (47.3)	1 REFERENCE	1 REFERENCE
Female	27,705 (61.5)	18,931 (64.4)	2103 (69.9)	6671 (52.7)	1.48 (1.32, 1.65)	1.38 (1.22, 1.55)
Age Group (years)						
65–74	7574 (16.8)	5689 (19.4)	239 (7.9)	1646 (13.0)	1 REFERENCE	1 REFERENCE
75–84	18,640 (41.4)	12,904 (43.9)	1060 (35.2)	4676 (37.0)	2.00 (1.63, 2.46)	1.84 (1.48, 2.29)
85–94	17,315 (38.4)	10,114 (34.4)	1516 (50.4)	5685 (44.9)	3.05 (2.49, 3.72)	2.53 (2.02, 3.16)
95+	1517 (3.4)	675 (2.3)	195 (6.5)	647 (5.1)	4.48 (3.39, 5.91)	3.33 (2.44, 4.54)
Ethnicity						
Māori	2487 (5.5)	1703 (5.8)	64 (2.1)	720 (5.7)	0.30 (0.20, 0.44)	0.37 (0.25, 0.56)
Pacifika	1430 (3.2)	1033 (3.5)	36 (1.2)	361 (2.9)	0.35 (0.22, 0.55)	0.48 (0.30, 0.77)
Asian	1037 (2.3)	755 (2.6)	52 (1.7)	230 (1.8)	0.38 (0.22, 0.64)	0.38 (0.22, 0.66)
European	39,732 (88.2)	25,628 (87.2)	2832 (94.1)	11,272 (89.1)	1 REFERENCE	1 REFERENCE
Other	360 (0.8)	263 (0.9)	26 (0.9)	71 (0.6)	1.29 (0.78, 2.11)	1.40 (0.85, 2.32)
Living Arrangement						
Lives alone	22,423 (49.8)	14,750 (50.2)	1476 (49.0)	6197 (49.0)	1 REFERENCE	1 REFERENCE
Lives with others	22,623 (50.2)	14,632 (49.8)	1534 (51.0)	6457 (51.0)	0.98 (0.88, 1.08)	0.99 (0.89, 1.09)

^a^2 values missing

Table 2 presents frequency distributions for each variable of interest, and unadjusted and adjusted SHR for each variable. Risk factors for hip fracture identified in the adjusted model from the variables listed in Additional file 1: Table S1 are sex, age, ethnicity, previous falls, previous hip fracture, wandering, low BMI, smoking tobacco, and Parkinson’s disease. Having shortness of breath, and, high BMI were related to a reduced risk of hip fracture. Having a previous hip fracture appeared to have the highest risk of sustaining a hip fracture with an adjusted SHR of 4.16.

**Table 2:** Variables of interest frequencies and subhazard ratios

		First Event				
Variable names	Total *n* (%)	Alive, no fracture	Fracture	Death	Crude SHR (95% CI)	Adjusted*
Cognitive Skills^b^						
Independent	21,505 (47.7)	14,815 (50.4)	1239 (41.2)	5451 (43.1)	1 REFERENCE	1 REFERENCE
Minimal Independence	16,512 (36.7)	10,651 (36.3)	1169 (38.8)	4692 (37.1)	1.32 (1.18, 1.48)	1.13 (1.00, 1.28)
Moderate to Severe dependence	7028 (15.6)	3915 (13.3)	602 (20.0)	2511 (19.8)	1.53 (1.33, 1.75)	1.16 (0.97, 1.41)
Hearing^c^						
Adequate	23,142 (51.4)	15,912 (54.2)	1390 (46.2)	5840 (46.2)	1 REFRENCE	1 REFERENCE
Minimal to moderate	20,056 (44.5)	12,518 (42.6)	1456 (48.4)	6082 (48.1)	1.19 (1.07, 1.32)	0.99 (0.88, 1.11)
Severe to none	1841 (4.1)	951 (3.2)	164 (5.4)	726 (5.7)	1.37 (1.08, 1.74)	0.99 (0.77, 1.55)
Vision^d^						
Adequate	32,013 (71.1)	21,552 (73.4)	1995 (66.3)	8466 (66.9)	1 REFERENCE	1 REFERENCE
Minimal to moderate	11,863 (26.3)	7165 (24.4)	919 (30.5)	3779 (29.9)	1.32 (1.19, 1.48)	1.14 (1.01, 1.28)
Severe to none	1162 (2.6)	663 (2.3)	96 (3.2)	403 (3.2)	1.38 (1.04, 1.83)	1.16 (0.87, 1.55)
Walking^a^						
Independent	34,524 (76.6)	24,553 (83.6)	1881 (62.5)	8090 (63.9)	1 REFERENCE	1 REFERENCE
Some assistance required	6633 (14.7)	3270 (11.1)	733 (24.4)	2630 (20.8)	1.40 (1.23, 1.60)	1.07 (0.79, 1.44)
Maximum Assistance/Dependent	2336 (5.2)	903 (3.1)	285 (9.5)	1148 (9.1)	1.15 (0.92, 1.45)	1.17 (0.71, 1.93)
Locomotion^e^						
Independent	35,033 (77.8)	24,905(84.8)	1919 (63.8)	8209 (64.9)	1 REFERENCE	1 REFERENCE
Some assistance required	6228 (13.8)	3051 (10.4)	683 (22.7)	2494 (19.7)	1.44 (1.26, 1.64)	1.08 (0.79, 1.47)
Dependent	2971 (6.6)	1118 (3.8)	335 (11.1)	1518 (12.0)	1.00 (0.80, 1.23)	0.94 (0.57, 1.55)
Primary Mode of Locomotion^b^						
Walking, no assistive device	14,486 (32.2)	11,155 (38.0)	498 (16.5)	2833 (22.4)	1 REFERENCE	1 REFERENCE
Assisted walking	28,367 (63.0)	17,290 (58.8)	2360 (78.4)	8717 (68.9)	1.29 (1.15, 1.45)	1.11 (0.97, 1.27)
Unable to walk	2192 (4.9)	936 (3.2)	152 (5.0)	1104 (8.7)	0.54 (0.39, 0.77)	0.71 (0.39, 1.27)
Timed 4 Metre walk^f^						
0–15 s	27,062 (60.1)	19,485 (66.3)	1551 (51.5)	6026 (47.6)	1 REFERENCE	1 REFERENCE
16–29 s	3927 (8.7)	2473 (8.4)	313 (10.4)	1141 (9.0)	1.09 (0.91, 1.30)	1.00 (0.84, 1.20)
30+ seconds	4026 (8.9)	2466 (8.4)	311 (10.3)	1249 (9.9)	1.14 (0.97, 1.35)	1.05 (0.88, 1.25)
Incomplete test	10,025 (22.3)	4955 (16.9)	835 (27.7)	4235 (33.5)	0.89 (0.78, 1.02)	0.88 (0.74, 1.03)
Total hours of exercise or physical activity^b^						
None/Less than 1 h	23,871 (53.0)	14,438 (49.1)	1612 (53.6)	7821 (61.8)	1 REFERENCE	1 REFERENCE
1–4 h	18,745 (41.6)	13,155 (44.8)	1267 (42.1)	4323 (34.2)	1.05 (0.95, 1.17)	1.05 (0.94, 1.17)
4 h or more	2429 (5.4)	1788 (6.1)	131 (4.4)	510 (4.0)	1.02 (0.82, 1.27)	1.12 (0.89, 1.40)
Number of days left house in last 3 days^b^						
None	14,987 (33.3)	7563 (25.7)	1371 (45.5)	6053 (47.8)	1 REFERENCE	1 REFERENCE
1–2 days	11,963 (26.6)	8011 (27.3)	782 (26.0)	3170 (25.1)	1.04 (0.92, 1.19)	1.18 (1.02, 1.36)
3 days	18,095 (40.2)	13,807 (47.0)	857 (28.5)	3431 (27.1)	0.83 (0.73, 0.94)	1.03 (0.88, 1.20)
Bladder Continence^g^						
Continent	28,200 (62.6)	19,125 (65.1)	1720 (57.1)	7355 (58.1)	1 REFERENCE	1 REFERENCE
Infrequently incontinent	4280 (8.5)	2819 (9.6)	286 (9.5)	1175 (9.3)	1.03 (0.86, 1.24)	0.93 (0.77, 1.11)
Occasionally incontinent	4079 (9.1)	2558 (8.7)	325 (10.8)	1196 (9.5)	1.30 (1.10, 1.54)	1.09 (0.92, 1.30)
Frequently Incontinent	6865 (15.2)	4161 (14.2)	541 (18.0)	2163 (17.1)	1.22 (1.06, 1.40)	1.07 (0.91, 1.25)
Incontinent	1586 (3.5)	705 (2.4)	133 (4.4)	748 (5.9)	1.06 (0.80, 1.39)	1.25 (0.90, 1.74)
Bowel Continence^h^						
Continent	37,767 (83.8)	25,665 (87.3)	2403 (79.8)	9699 (76.6)	1 REFERENCE	1 REFERENCE
Infrequently incontinent	2912 (6.5)	1710 (5.8)	211 (7.0)	991 (7.8)	1.14 (0.91, 1.36)	0.99 (0.80, 1.23)
Occasionally Incontinent	2331 (5.2)	1159 (3.9)	221 (7.3)	951 (7.5)	1.34 (1.09, 1.64)	1.10 (0.87, 1.39)
Frequently Incontinent	1089 (2.4)	475 (1.6)	94 (3.1)	520 (4.1)	0.95 (0.68, 1.34)	0.82 (0.56, 1.20)
Incontinent	840 (1.9)	326 (1.1)	73 (2.4)	441 (3.5)	0.67 (0.42, 1.06)	0.87 (0.49, 1.56)
Fatigue^i^						
None	13,194 (29.3)	9642 (32.8)	786 (26.1)	2766 (21.9)	1 REFERENCE	1 REFERENCE
Minimal to Moderate	26,380 (58.6)	17,224 (58.6)	1897 (63.0)	7259 (57.4)	1.19 (1.05, 1.33)	1.16 (1.02, 1.31)
Severe	5469 (12.1)	2514 (8.6)	327 (10.9)	2628 (20.8)	0.89 (0.74, 1.07)	0.88 (0.70, 1.09)
Difficult or unable to move self to standing^i^						
Not present	28,012 (62.2)	19,616 (66.8)	1619 (53.8)	6777 (53.6)	1 REFERENCE	1 REFERENCE
Present	17,031 (37.8)	9764 (33.2)	1391 (46.2)	5876 (46.4)	1.03 (0.93, 1.15)	0.98 (0.86, 1.11)
Dizziness^i^						
Not present	38,108 (84.6)	24,968 (85.0)	2565 (85.2)	10,575 (83.6)	1 REFERENCE	1 REFERENCE
Present	6935 (15.4)	4412 (15.0)	445 (14.8)	2078 (16.4)	1.06 (0.92, 1.21)	1.07 (0.92, 1.23)
Unsteady Gait^i^						
Not present	21,569 (47.9)	15,058 (51.2)	1187 (39.4)	5324 (42.1)	1 REFERENCE	1 REFERENCE
Present	23,474 (52.1)	14,322 (48.7)	1823 (60.6)	7329 (57.9)	1.12 (1.01, 1.24)	1.02 (0.90, 1.16)
Previous Fall^i^						
No Fall	26,889 (59.7)	18,626 (63.4)	1199 (39.8)	7064 (55.8)	1 REFERENCE	1 REFERENCE
Had at least one fall	18,154 (40.3)	10,754 (36.6)	1811 (60.2)	5589 (44.2)	1.36 (1.23, 1.51)	1.17 (1.05, 1.31)
Previous hip fracture^a^						
None	44,232 (98.2)	29,266 (99.6)	2369 (78.7)	12,567 (99.5)	1 REFERENCE	1 REFERENCE
Had previous fracture	812 (1.8)	115 (0.4)	641 (21.3)	56 (0.4)	5.37 (3.83, 7.52)	4.16 (2.93, 5.89)
Previous Other fracture^a^						
None	43,704 (97.0)	28,629 (97.4)	2816 (93.6)	12,259 (96.9)	1 REFERENCE	1 REFERENCE
Had previous fracture	1340 (3.0)	752 (2.6)	194 (6.4)		1.55 (1.20, 2.01)	1.03 (0.78, 1.36)
Easily Distracted^j^						
Not present	34,633 (76.9)	22,806 (77.6)	2250 (74.8)	9577 (75.7)	1 REFERENCE	1 REFERENCE
Present	10,400 (23.1)	6572 (22.4)	760 (25.2)	3068 (24.2)	1.34 (1.20, 1.50)	1.11 (0.96, 1.27)
Mental Function Varies over the course of a day^j^						
Not present	35,107 (77.9)	23,404 (79.7)	2259 (75.0)	9444 (74.60	1 REFERENCE	1 REFERENCE
Present	9926 (22.0)	5974 (20.3)	751 (25.0)	3201 (25.3)	1.39 (1.24, 1.56)	1.16 (1.00, 1.33)
Wandering^k^						
Not Present	43,254 (96.0)	28,287 (96.3)	2867 (95.2)	12,100 (95.6)	1 REFERENCE	1 REFERENCE
Present	1783 (4.0)	1092 (3.7)	143 (4.8)	548 (4.3)	1.67 (1.36, 2.06)	1.36 (1.07, 1.72)
Frequency of Pain^i^						
No pain	18,291 (40.6)	11,660 (39.7)	1093 (36.3)	5538 (43.8)	1 REFERENCE	1 REFERENCE
Not in last 3 days	4573 (10.2)	3054 (10.4)	324 (10.8)	1195 (9.4)	1.41 (0.96, 1.35)	1.34 (0.99, 1.81)
At least once in last 3 days	22,179 (49.2)	14,666 (49.9)	1593 (52.9)	5920 (46.8)	0.91 (0.82, 1.01)	1.11 (0.81, 1.53)
Intensity of Highest level of Pain^i^						
None	18,495 (41.1)	11,759 (40.0)	1121 (37.2)	5615 (44.4)	1 REFERENCE	1 REFERENCE
Mild to Moderate	19,860 (44.1)	13,091 (44.6)	1502 (49.9)	5267 (41.6)	0.92 (0.83, 1.03)	0.69 (0.50, 0.96)
Severe to Excruciating	6688 (14.8)	4530 (15.4)	387 (12.9)	1771 (14.0)	0.91 (0.77, 1.06)	0.75 (0.52, 1.07)
Consistency of Pain^i^						
None/Very Little	19,792 (43.9)	12,635 (43.0)	1193 (39.6)	5964 (47.1)	1 REFERENCE	1 REFERENCE
Intermittent	19,399 (43.1)	12,748 (43.4)	1478 (49.1)	5173 (40.9)	0.96 (0.86, 1.07)	1.22 (0.93, 1.58)
Constant	5852 (13.0)	3997 (13.6)	339 (11.3)	1516 (12.0)	0.89 (0.76, 1.05)	1.24 (0.92, 1.68)
Body Mass Index^m^						
Underweight	2292 (5.1)	1137 (3.9)	305 (10.1)	850 (6.7)	1.87 (1.56, 2.24)	1.67 (1.39, 2.02)
Normal	13,538 (30.1)	8714 (29.7)	1004 (33.4)	3820 (30.2)	1 REFERENCE	1 REFERENCE
Overweight	7480 (16.6)	5433 (18.5)	329 (10.9)	1718 (13.6)	0.58 (0.49, 0.69)	0.66 (0.55, 0.80)
Obese	4616 (10.2)	3630 (12.4)	132 (4.4)	854 (6.7)	0.33 (0.25, 0.44)	0.47 (0.36, 0.63)
Smokes tobacco daily^i^						
No	42,644 (94.7)	27,851 (94.8)	2841 (94.4)	11,952 (94.5)	1 REFERENCE	1 REFERENCE
Yes	2399 (5.3)	1529 (5.2)	169 (5.6)	701 (5.5)	1.21 (0.98, 1.49)	1.56 (1.25, 1.96)
Consumes Alcohol^i^						
None	35,914 (79.7)	22,947 (78.1)	2539 (84.4)	10,428 (82.4)	1 REFERENCE	1 REFERENCE
At least one drink	9129 (20.3)	6433 (21.9)	471 (15.6)	2225 (17.6)	1.22 (1.06, 1.39)	0.90 (0.78, 1.03)
Weight Loss of 5% or more^i^						
No	38,317 (85.1)	25,966 (88.4)	2433 (80.8)	9918 (78.4)	1 REFERENCE	1 REFERENCE
Yes	6726 (14.9)	3414 (11.6)	577 (19.2)	2735 (21.6)	1.06 (0.90, 1.25)	1.08 (0.93, 1.26)
Dehydrated^i^						
No	44,175 (98.1)	29,001 (98.7)	2933 (97.4)	12,241 (96.7)	1 REFERENCE	1 REFERENCE
Yes	868 (1.9)	379 (1.3)	77 (2.6)	412 (3.3)	1.15 (0.81, 1.63)	0.82 (0.57, 1.19)
Decrease in food/fluid consumed^i^						
No	40,280 (89.4)	27,041 (92.0)	2649 (88.0)	10,590 (83.7)	1 REFERENCE	1 REFERENCE
Yes	4763 (10.6)	2339 (8.0)	361 (12.0)	2063 (16.3)	1.06 (0.90, 1.25)	0.95 (0.78, 1.14)
Parkinson’s Disease^a^						
Not present	43,263 (96.0)	28,229 (96.1)	2854 (94.8)	12,180 (96.3)	1 REFERENCE	1 REFERENCE
Diagnosis present	1781 (4.0)	1152 (3.9)	156 (5.2)	473 (3.7)	1.38 (1.10, 1.72)	1.45 (1.14, 1.84)
Stroke/CVA^a^						
Not Present	37,121 (82.4)	24,339 (82.8)	2527 (84.0)	10,255 (81.0)	1 REFERENCE	1 REFERENCE
Diagnosis Present	7923 (17.6)	5042 (17.2)	483 (16.0)	2398 (19.0)	0.87 (0.75, 1.00)	0.90 (0.78, 1.04)
COPD^a^						
Not present	37,920 (84.2)	25,293 (86.1)	2578 (85.6)	10,049 (79.4)	1 REFERENCE	1 REFERENCE
Diagnosis present	7124 (15.8)	4088 (13.9)	432 (14.4)	2604 (20.6)	0.92 (0.79, 1.06)	1.15 (0.98, 1.35)
Dyspnoea^i^						
Not present	24,021 (53.3)	16,555 (56.3)	1772 (58.9)	5694 (45.0)	1 REFERENCE	1 REFERENCE
Present	21,022 (46.7)	12,825 (43.6)	1238 (41.1)	6959 (55.0)	0.79 (0.71, 0.88)	0.80 (0.71, 0.90)
Environment^d^						
No	39,487 (87.7)	25,908 (88.2)	2615 (86.9)	10,964 (86.6)	1 REFERENCE	1 REFERENCE
Yes	5551 (12.3)	3468 (11.8)	395 (13.1)	1688 (13.3)	1.05 (0.90, 1.22)	1.11 (0.89, 1.09)

^a^2 values missing

^b^1 value missing

^c^7 values missing

^d^8 values missing

^e^814 values missing

^f^6 variables missing

^g^36 values missing

^h^107 values missing

^i^3 values missing

^j^13 values missing

^k^9 values missing

^m^17,120 values missing

*Adjusted for age, sex and ethnicity

### Sex differences

Table 3 provides a comparison of the adjusted models between males and females. Significant risk factors for each group are marked in bold. There were differences in significant risk factors for males and females. The significant risk factors identified in the model for females were age, ethnicity, falls, previous hip fractures, wandering, BMI, tobacco use and shortness of breath. The significant factors identified for males were age, previous hip fracture, Parkinson’s disease and shortness of breath.

**Table 3:** Comparison of risk factors for hip fracture between males and females

Variable names	Males Adjusted Analysis SHR (95% CI)	Females Adjusted Analysis SHR (95% CI)
Age Group (years)		
65–74	**1 REFERENCE**	**1 REFERENCE**
75–84	**2.12 (1.48, 3.04)**	**1.70 (1.30, 2.23)**
85–94	**2.46 (1.68, 3.60)**	**2.51 (1.90, 3.31)**
95+	**3.48 (1.88, 6.42)**	**3.22 (2.23, 4.66)**
Ethnicity		
Māori	0.36 (0.16, 0.81)	**0.37 (0.23, 0.60)**
Pacifika	0.55 (0.24, 1.25)	**0.45 (0.25, 0.80)**
Asian	0.58 (0.26, 1.29)	**0.30 (0.14, 0.64)**
European	1 REFERENCE	**1 REFERENCE**
Other	1.78 (0.78, 4.06)	**1.24 (0.65, 2.34)**
Living Arrangement		
Lives alone	1 REFERENCE	1 REFERENCE
Lives with others	0.84 (0.69, 1.01)	1.05 (0.93, 1.19)
Cognitive Skills		
Independent	1 REFERENCE	1 REFERENCE
Minimal Independence	1.18 (0.94, 1.48)	1.10 (0.95, 1.29)
Moderate to Severe dependence	1.07 (0.75, 1.52)	1.21 (0.97, 1.50)
Hearing		
Adequate	1 REFERENCE	1 REFERENCE
Minimal to moderate	1.04 (0.85, 1.28)	0.97 (0.84, 1.11)
Severe to none	1.20 (0.80, 1.80)	0.91 (0.66, 1.25)
Vision		
Adequate	1 REFERENCE	1 REFERENCE
Minimal to moderate	1.14 (0.92, 1.41)	1.13 (0.99, 1.30)
Severe to none	1.13 (0.64, 1.99)	1.16 (0.82, 1.62)
Walking		
Independent	1 REFERENCE	1 REFERENCE
Some assistance required	0.97 (0.58, 1.62)	1.11 (0.76, 1.61)
Maximum Assistance/Dependent	0.70 (0.34, 1.45)	1.52 (0.80, 2.87)
Locomotion		
Independent	1 REFERENCE	1 REFERENCE
Some assistance required	1.13 (0.67, 1.90)	1.05 (0.72, 1.55)
Dependent	1.54 (0.78, 3.04)	0.75 (0.38, 1.45)
Primary Mode of Locomotion		
Walking, no assistive device	1 REFERENCE	1 REFERENCE
Assisted walking	1.12 (0.88, 1.44)	1.10 (0.94, 1.30)
Unable to walk	0.68 (0.25, 1.88)	0.73 (0.35, 1.51)
Timed 4 Metre walk		
0–15 s	1 REFERENCE	1 REFERENCE
16–29 s	0.91 (0.65, 1.28)	1.04 (0.84, 1.29)
30+ seconds	1.02 (0.74, 1.40)	1.07 (0.87, 1.32)
Incomplete test	0.75 (0.55, 1.01)	0.93 (0.77, 1.13)
Total hours of exercise or physical activity		
None/Less than 1 h	1 REFERENCE	1 REFERENCE
1–4 h	1.07 (0.86, 1.32)	1.04 (0.91, 1.19)
4 h or more	0.88 (0.56, 1.39)	1.21 (0.93, 1.58)
Number of days left house in last 3 days		
None	1 REFERENCE	1 REFERENCE
1–2 days	1.29 (0.98, 1.70)	1.13 (0.85, 1.34)
3 days	1.01 (0.76, 1.35)	1.03 (0.86, 1.23)
Bladder Continence		
Continent	1 REFERENCE	1 REFERENCE
Infrequently incontinent	0.85 (0.58, 1.24)	0.96 (0.77, 1.19)
Occasionally incontinent	1.02 (0.72, 1.45)	1.12 (0.91, 1.37)
Frequently Incontinent	0.87 (0.62, 1.22)	1.13 (0.95, 1.36)
Incontinent	1.57 (0.95, 2.61)	1.11 (0.72, 1.72)
Bowel Continence		
Continent	1 REFERENCE	1 REFERENCE
Infrequently incontinent	0.86 (0.57, 1.29)	1.07 (0.83, 1.37)
Occasionally Incontinent	0.99 (0.63, 1.54)	1.16 (0.88, 1.53)
Frequently Incontinent	0.66 (0.32, 1.37)	0.92 (0.58, 1.44)
Incontinent	0.96 (0.42, 2.24)	0.77 (0.35, 1.74)
Fatigue		
None	1 REFERENCE	1 REFERENCE
Minimal to Moderate	1.32 (1.04, 1.69)	1.10 (0.95, 1.27)
Severe	1.25 (0.85, 1.83)	0.74 (0.56, 0.98)
Difficult or unable to move self to standing		
Not present	1 REFERENCE	1 REFERENCE
Present	0.94 (0.75, 1.18)	1.00 (0.85, 1.17)
Dizziness		
Not present	1 REFERENCE	1 REFERENCE
Present	1.09 (0.84, 1.41)	1.07 (0.89, 1.27)
Unsteady Gait		
Not present	1 REFERENCE	1 REFERENCE
Present	1.05 (0.83, 1.33)	1.01 (0.87, 1.17)
Previous Fall		
No Fall	1 REFERENCE	**1 REFERENCE**
Had at least one fall	1.20 (0.98, 1.47)	**1.15 (1.01, 1.32)**
Previous hip fracture		
None	**1 REFERENCE**	**1 REFERENCE**
Had previous fracture	**4.45 (1.95, 10.13)**	**4.05 (2.75, 5.95)**
Previous Other fracture		
None	1 REFERENCE	1 REFERENCE
Had previous fracture	1.43 (0.78, 2.60)	0.96 (0.70, 1.32)
Easily Distracted		
Not present	1 REFERENCE	1 REFERENCE
Present	1.06 (0.82, 1.38)	1.12 (0.95, 1.32)
Mental Function Varies over the course of a day		
Not present	1 REFERENCE	1 REFERENCE
Present	1.25 (0.97, 1.61)	1.12 (0.94, 1.32)
Wandering		
Not Present	1 REFERENCE	**1 REFERENCE**
Present	1.13 (0.72, 1.75)	**1.48 (1.12, 1.98)**
Frequency of Pain		
No pain	1 REFERENCE	1 REFERENCE
Not in last 3 days	1.41 (0.85, 2.36)	1.31 (0.90, 1.89)
At least once in last 3 days	1.42 (0.83, 2.45)	1.02 (0.69, 1.50)
Intensity of Highest level of Pain		
None	1 REFERENCE	1 REFERENCE
Mild to Moderate	0.62 (0.35, 1.08)	0.73 (0.49, 1.09)
Severe to Excruciating	0.72 (0.38, 0.72)	0.77 (0.50, 1.21)
Consistency of Pain		
None/Very Little	1 REFERENCE	1 REFERENCE
Intermittent	0.94 (0.62, 1.45)	1.33 (0.95, 1.85)
Constant	0.94 (0.55, 1.59)	1.37 (0.94, 1.99)
Body Mass Index		
Underweight	1.55 (0.98, 2.47)	**1.69 (1.37, 2.09)**
Normal	1 REFERENCE	**1 REFERENCE**
Overweight	0.81 (0.61, 1.07)	**0.58 (0.46, 0.74)**
Obese	0.49 (0.29, 0.83)	**0.47 (0.33, 0.65)**
Smokes tobacco daily		
No	1 REFERENCE	**1 REFERENCE**
Yes	1.33 (0.88, 2.00)	**1.70 (1.30, 2.23)**
Consumes Alcohol		
None	1 REFERENCE	1 REFERENCE
At least one drink	0.83 (0.66, 1.05)	0.93 (0.78, 1.11)
Weight Loss of 5% or more		
No	1 REFERENCE	1 REFERENCE
Yes	1.27 (0.97, 1.66)	1.01 (0.84, 1.21)
Dehydrated		
No	1 REFERENCE	1 REFERENCE
Yes	0.54 (0.24, 1.22)	0.95 (0.63, 1.45)
Decrease in food/fluid consumed		
No	1 REFERENCE	1 REFERENCE
Yes	0.92 (0.65, 1.30)	0.96 (0.77, 1.19)
Parkinson’s Disease		
Not present	**1 REFERENCE**	1 REFERENCE
Diagnosis present	**1.50 (1.08, 2.09)**	1.37 (0.96, 1.96)
Stroke/CVA		
Not Present	1 REFERENCE	1 REFERENCE
Diagnosis Present	1.05 (0.83, 1.34)	0.83 90.69, 1.00)
COPD		
Not present	1 REFERENCE	**1 REFERENCE**
Diagnosis present	1.19 (0.90, 1.57)	**0.82 (0.71, 0.94)**
Dyspnoea		
Not present	**1 REFERENCE**	**1 REFERENCE**
Present	**0.77 (0.61, 0.96)**	**1.12 (0.92, 1.37)**
Environment		
No	1 REFERENCE	1 REFERENCE
Yes	1.02 (0.77, 1.37)	1.15 (0.95, 1.38)

Models adjusted for age and ethnicity

Bolded variables are variables that are statistically significant

## Discussion

### Key findings

This study identified risk factors for hip fracture within a community dwelling cohort, and stratified by sex. To our knowledge this is the first study in New Zealand that has examined a large suite of risk factors associated with hip fractures amongst older adults in New Zealand. Risk factors identified in this study were age, female sex, ethnicity, falls, previous hip fracture, wandering, tobacco use, low BMI, shortness of breath and Parkinson’s disease.

Sex differences in risk were found between males and females. Significant risk factors for males were age, previous hip fracture, and Parkinson’s disease. Females had more significant risk factors than males including age, previous hip fracture, ethnicity, falls, wandering, BMI, tobacco use, and shortness of breath. For the female group a diagnosis of chronic obstructive pulmonary disease (COPD) was associated with a reduced risk of hip fracture.

### Findings within the literature

Several of the findings within this study are consistent with previous research. For example, falls are consistently deemed a significant risk for hip fracture. Most hip fractures in older people occur from a fall [33]. Similarly, prior studies on hip fracture risk have found previous hip fractures to be a significant risk factor [21, 34, 35]. Within this study, previous hip fractures have been identified as having the largest risk of sustaining a hip fracture (SHR 4.16, *p* < 0.001). People who have had a previous hip fracture are around four times more likely to have a second hip fracture compared to individuals who have not had a prior fracture. This suggests emphasis should be placed on preventing a subsequent hip fracture in people who have already had a previous hip fracture. Additionally, those with a low BMI are more likely to have a hip fracture than those who have a normal BMI, and those overweight or obese had a lowered risk for hip fracture. These findings are consistent with the literature [11, 36–38]. Our study did not find gait speed to be a significant risk factor for hip fracture, however a recent study by Harvey et al. found greater walking speed significantly reduced the risk of hip fracture [39].

Demographic differences such as age, gender and ethnicity are all commonly known risk factors for hip fracture [2]. As age increases the likelihood of a hip fracture increases. Females are known to have a higher risk of hip fracture than males as they are more likely to develop osteoporosis and have lower bone mineral density. In this study, females had a higher number of significant risk factors than did men, suggesting there are more risks common to females. People with different ethnic backgrounds have differing risks for hip fracture in the current study and in previous hip fracture risk studies [6, 40, 41]. The level of risk for hip fracture in Māori and Pasifika people has not been researched recently, however, an earlier New Zealand study noted that Māori males are less likely to have a hip fracture than non-Māori and female Māori [17]. In the current study after adjusting for age and sex, Māori (SHR 0.37, *p* < 0.001) and Asian (SHR 0.38, *p* < 0.001) participants had the lowest risk of hip fracture, followed by Pasifika (SHR 0.48, *p* < 0.001) participants; individuals who were classified as other ethnicities had the highest risk of fracture but the group was small and diverse therefore, no substantial conclusions can be made about that group. Earlier research on ethnic differences for hip fracture found that individuals of Polynesian descent, including Pasifika people, tend to be less at risk of hip fracture than European individuals due to a higher BMD [16]. Reid et al. found there was no difference in BMD between Māori and Polynesian individuals [42]. This is consistent with our study, which found that Māori and Pasifika have a lower risk of hip fracture than European individuals. In addition to ethnic differences in BMD, there may be differences in other parameters of bone strength that contribute to the observed ethnic variation in fracture rates. For example, prior literature shows that Asian people tend to have a reduced risk of hip fracture as compared with people of European descent despite having lower BMI on average [41], which may be explained by shorter femoral necks among Asian people [16]. Further research to identify ethnic-specific risk factors may be beneficial.

Tobacco use has been associated with hip fracture risk in previous studies [7, 43, 44]. A study by Kanis et al. determined that non-smokers had the lowest risk of sustaining a hip fracture and current smokers had a higher risk of hip fracture than people who no longer smoke [43]. Research conducted around recovery after hip fracture suggests that smokers have longer recovery times and more complications after a fracture [45, 46].

Literature on hip fracture risk suggests that Parkinson’s disease is a potential risk for hip fracture [47]. Our study found that, Parkinson’s disease was significantly associated with hip fracture risk. People with a diagnosis of Parkinson’s disease were more likely to sustain a hip fracture than those with no diagnosis (SHR 1.45, *p* < 0.05). Individuals with Parkinson’s disease have a higher risk of falling and thus are more likely to have an increased risk of hip fracture [2, 47, 48].

Shortness of breath was associated with a reduced risk of hip fracture. This variable has not explicitly been studied in the literature. People who have shortness of breath are potentially less likely to be involved in activities that can lead to hip fracture.

There have been three other known studies identified that look specifically at hip fracture risk in a home care population. The first paper, from Canada, found risk factors for hip fracture to be female gender, older age, osteoporosis, falls, unsteady gait, use of ambulation aid, tobacco use, severe malnutrition and cognitive impairment. Arthritis and morbid obesity were found to be protective factors [7]. Our study also found female gender, older age, falls and tobacco use to be significant. Risk factors that were significant in our model but not in the Canadian model were prior hip fracture, shortness of breath, BMI, and wandering. Differences between the models could be due to the different statistical techniques used, the different version of assessment or the different nationalities of the cohorts. Canada uses the MDS, an older version of the assessment used in New Zealand, and so not all questions are the same. For instance, osteoporosis is not assessed in the New Zealand interRAI-HC so its associated risk could not be determined. The other two studies, from New Zealand, looked at specific variables, the first study found that urinary incontinence was an independent risk factor for falls but not for hip fractures [18]. Our study also found urinary incontinence was non-significant. The second study, found that medications specific to the drug burden index (DBI) were significant for hip fracture risk [14]. While the interRAI-HC contains some medications, the list is not comprehensive and medications were omitted from this analysis.

### Limitations of the study

There are well-known risk factors for hip fracture that could not be explored using the interRAI -HC assessment. For example, the interRAI version used for this study does not contain osteoporosis as a diagnosis, therefore those with osteoporosis may be at a higher risk of hip fracture but they are unable to be identified within the cohort. Osteoporosis is a known-risk factor for hip fracture as it causes weakening of the bones, leading to a higher probability of fracture [2]. Another common risk factor for hip fracture is low BMD, however, determining the level of BMD in a person requires them to undergo testing in a hospital [49]. As those requiring a home care assessment are usually more frail than the typical population of older adults, measuring BMD can be burdensome [10]. Identifying risk factors within the interRAI-HC assessment initially, for people who are frail can be used as a way of identifying people at an elevated risk of fracture. The FRAX [50] and Garvan [51, 52] scores are commonly used in assessing a person’s hip fracture risk. However, both scores are calculated in a clinical setting which would require the individual to visit their general practitioner to carry out the assessment. As those undergoing a home care assessment are generally frailer than the general population of older people, it may be easier to estimate fracture risk as part of their interRAI assessment.

Within the interRAI-HC BMI recordings are low with around 40% of individuals having no recorded BMI [24]. This is largely due to it being difficult to measure height and weight data for some of the more frail older people, particularly those who are bed or wheelchair bound. However, the results from the known BMI’s were significant which suggests BMI is an important factor in determining a person’s hip fracture risk.

This study was conducted using New Zealand data and may not be generalizable to a wider international audience, however the results may be generalizable to other home care audiences. The interRAI-HC assesses the health needs of people who have complex issues and may not be generalizable to a healthier cohort of people aged 65+ years.

### Summary of the implications of the work for practice and research

The New Zealand healthy ageing strategy aims to support people with high and complex needs, such as those using home care services, to ensure they can live as independently as possible [53]. The interRAI-HC is useful for assessing the needs of these people to ensure appropriate care is implemented. Individuals receiving home care have different needs to a general population of older adults [7]. Hip fractures cause significant disability for many older adults, with some of the worst outcomes for those aged 65 years or older. Now that risk factors for hip fracture have been identified for community-dwelling older people; further work can be done to create a risk score to identify those who are at a relatively high risk of hip fracture using the questions within the interRAI-HC assessment. Further work will be done using these variables to develop a prediction model to the randomly omitted data to determine their psychometric utility in screening for hip fracture risk.

## Conclusions

Falls, previous hip fractures, being underweight, older age, female gender, prone to wandering, a diagnosis of Parkinson’s disease, and being a smoker all contribute to hip fracture risk. Being overweight, and having shortness of breath can decrease the risk of hip fracture. Individuals who are not of European ethnicity were found to have a reduced risk of hip fracture. These risk factors agree with international work on risk factors for hip fracture and in the future this information could be used to predict hip fracture risk in older people as a routine part of their health assessment.

## Additional file

Additional file 1: Supplementary Materials for Risk Factors for hip fracture in New Zealand older adults seeking home care services – A national population cross-sectional study. **Table S1.** is a record of the variables used for analysis from questions based on the interRAI-HC version 9.1 with New Zealand specifications. The table contains information on the question number and how the questions were recoded for the purposes of analysis. (DOCX 21 kb)

## Abbreviations

ARC: Aged Residential Care
BMD: Bone Mineral Density
BMI: Body Mass Index
CI: Confidence Interval
COPD: Chronic Obstructive Pulmonary Disease
CPS: Cognitive Performance Scale
DBI: Drug Burden Index
ICD-10 AM: International Statistical Classification of Diseases and Related Health Problems, Tenth Revision, Australian Modification
interRAI-HC: international Residential Assessment Instrument, Home Care
MDS: Minimum Dataset
MORT: Mortality Collection
NHI: National Health Index
NMDS: National Minimum Dataset
RECORD: REporting of Studies Conducted using Observational Routinely collected Data
SHR: Subhazard Ratio
STROBE: STrengthening the Reporting of OBservational studies in Epidemiology
TAS: Technical Advisory Services
