# Supplementary material for: Risk factors for hip fracture in New Zealand older adults seeking home care services: a national population cross-sectional study
Source: BMC Geriatr. 2019 Mar 25;19:93. doi: 10.1186/s12877-019-1107-1 (PMC6434861; doi:10.1186/s12877-019-1107-1)
Supplement: Supplementary file 1 — Supplementary Materials for Risk Factors for hip fracture in New Zealand older adults seeking home care services – A national population cross-sectional study. Table S1. is a record of the variables used for analysis from questions based on the interRAI-HC version 9.1 with New Zealand specifications. The table contains information on the question number and how the questions were recoded for the purposes of analysis. (DOCX 21 kb) [file 12877_2019_1107_MOESM1_ESM.docx]

**Supplementary Materials for Risk Factors for hip fracture in New Zealand older adults seeking home care services – A national population cross-sectional study**

Additional file 1: Table S1 is a record of the variables used for analysis from questions based on the interRAI-HC version 9.1 with New Zealand specifications. The table contains information on the question number and how the questions were recoded for the purposes of analysis.

**Additional file 1:** **Table S1 Variables used for analysis**

| **Domain** | **Question** | **iCode** | **How recoded** |
| --- | --- | --- | --- |
| **Demographics** | A2 Gender | iA2 | M = 0  F = 1  U = Missing  I = Missing |
|  | A3 Age Group | iA3 | 65-74 = 1  75-84 = 2  85-94 = 3  95+ = 4 |
|  | B2a-B2y  Ethnicity | MH_00005a -  MH_00005y | Maori = 1  Pacifika = 2  Asian = 3  European = 4  Other = 5 |
| **Cognitive/Functional** | C1 Cognitive skills for daily decision making | iC1 | 0 = 0 Independent  1,2 = 1 Minimal Independence  3, 4 = 2 Moderate to Severe  5 = 3 No discernible conscousness, coma |
|  | D3 Hearing | iD3a | 0 = 0 Adequate  1,2 = 1 Minimal to moderate  3,4 = 2 Severe to none |
|  | D4 Vision | iD4a | 0 = 0 Adequate  1,2 = 1 Minimal to moderate  3,4 = 2 Severe to none |
|  | G2e Walking | iG2e | 0,1 = 0 Independent  2, 3 = 1 Some assistance required  4,5,6 = 2 Maximum assistance/dependent  8 = Missing |
|  | G2f Locomotion | iG2f | 0,1 = 0 Independent  2,3 = 1 Some assistance  4,5,6 = 2 Maximum assistance/dependent  8 = Missing |
|  | G3a Primary mode of locomotion | iG3 | 0 = 0 Walking, no assistive device  1 = 1 Assisted walking  2,3 = 2 Cannot walk |
|  | G3b Timed 4 metre walk | iG12 | 1 0-15 seconds  2 16-29 seconds  3 30 or more seconds  77,88,99 = 4 Incomplete tests |
|  | G4a Total hours of exercise or physical activity | iG6a | 0,1 = 0 None/Less than 1 hour  2,3 = 1 1-4 hours  4 = 2 More than 4 hours |
|  | G4b In the last 3 days, number of days went out of the house | iG6b | 0,1 = 0 None  2 = 1 1-2 days  3 = 2 3 days |
|  | H1 Bladder Continence | iH1 | 0,1, - 1 Continent  2,3,4,5 leave as is  8 as missing |
|  | H3 Bowel Continence | iH3 | 0,1, - 1 Continent  2,3,4,5 leave as is  8 as missing |
|  | J5 Fatigue | iJ4 | 0 = 0 None  1,2 = 1 Minimal to moderate  3,4 = 2 Severe |
|  | J3a Difficult or unable to move self to a standing position | iJ2a | 0,1 = 0 Not present  2,3,4 = 1 Present |
|  | J3c Dizziness | iJ2c | 0,1 = 0 Not present  2,3,4 = 1 Present |
|  | J3d Unsteady Gait | iJ2d | 0,1 = 0 Not present  2,3,4 = 1 Present |
| **Falls and Fractures** |  |  |  |
|  | J1 Previous Fall | iJ1 | 0 = 0 No falls  1,2,3 = At least one fall |
|  | I1a Previous hip fracture during last 30 days | iI1a | 0 = 0 No fracture  1,2,3 = 1 Previous hip fracture |
|  | I1b Other fracture during last 30 days | iI1b | 0 = 0 No fracture  1,2,3 = 1 Had previous fracture |
| **Neuropsychiatric** |  |  |  |
|  | C3a Easily Distracted | iC3a | 0 = 0 Not present  1,2 = 1 Present |
|  | C3c Mental Function Varies | iC3c | 0 = 0 Not present  1 = 1 Present |
|  | E3a Wandering | iE3a | 0 = 0 Not present  1,2,3 = 1 Present |
| **Pain** |  |  |  |
|  | J6a Frequency of pain | iJ5a | 0 =0 No pain  1 =1 No pain in last 3 days  2,3 = 2 At least once in last 3 days |
|  | J6b Intensity of highest level of pain | iJ5b | 0 = 0 None  1,2 = 1 Mild to moderate  3,4 = Severe to excruciating |
|  | J6c Consistency of pain | iJ5c | 0,1 = 0 None to very little  2 = 1 Intermittent  3 = 2 Constant |
| **Nutrition** |  |  |  |
|  | BMI | Scale_BMI | 9- 18 Underweight  18 -25 Normal  26 – 30 Overweight  31 – 41 Obese  Missing/Unknown |
|  | J9a Smokes tobacco daily | iJ8a | 0 = 0 Non-smoker  1,2 = 1 Smoker |
|  | J9b Alcohol – highest number of drinks in any ‘single sitting’ | iJ8b | 0 = 0 None  1,2,3 = 1 At least 1 drink |
|  | K2a Weight loss of 5% or more | iK2a | 0 = 0 No  1 = 1 Yes |
|  | K2b Dehydrated | iK2c | 0 = 0 No  1 = 1 Yes |
|  | K2e Decrease in amount of food or fluid usually consumed | iK2g | 0 = 0 No  1 = 1 Yes |
| **Co-morbidities** |  |  |  |
|  | I1h Parkinson’s Disease | iI1h | 0 = 0 Not present  1,2,3 = 1 Diagnosis present |
|  | I1j Stroke/CVA | Ii1j | 0 = 0 Not present  1,2,3 = 1 Diagnosis present |
|  | I1L COPD | iI1L | 0 = 0 Not present  1,2,3 = 1 Diagnosis present |
|  | J4 Dyspnoea (Shortness of Breath) | iJ3 | 0 = 0 None  1,2 = 1 Absent at rest  3 = 2 Present at rest |
|  | J7c End-stage disease, 6 or fewer months left to live | iJ6c | 0 = 0 No  1 = 1 Yes |
